# First report of immature stages of *Ixodes bocatorensis* (Ixodida: Ixodidae) on small mammals in the Maracanã Environmental Protection Area, São Luís, Maranhão, Brazil

**DOI:** 10.1590/S1984-29612025028

**Published:** 2025-06-02

**Authors:** Francielma Chaves Sousa Gonçalves, Alana dos Santos Cardoso, Ana Karoline Sousa Mendes Simas, Débora Ellen Pinheiro Silva, Clauberth César Alves Carvalho, Davi Viegas Melo, Andréa Pereira da Costa, Rita de Maria Seabra Nogueira, Thiago Fernandes Martins, Marcelo Bahia Labruna, Francisco Borges Costa

**Affiliations:** 1 Laboratório de Parasitologia e Doenças Parasitárias, Departamento de Patologia, Universidade Estadual do Maranhão – UEMA, São Luís, MA, Brasil; 2 Departamento de Medicina Veterinária Preventiva e Saúde Animal, Faculdade de Medicina Veterinária e Zootecnia, Universidade de São Paulo – USP, São Paulo, SP, Brasil

**Keywords:** Ticks, wild animals, Amazon biome, Carrapatos, animais silvestres, bioma amazônico

## Abstract

Research into ticks that feed on small mammals is important because they can be vectors of pathogenic bioagents that infect animals and humans. In this study, small mammals were captured in the Maracanã Environmental Protection Area, in the eastern Amazon biome (São Luís, Maranhão), and were visually inspected to detect the presence of ectoparasites. Overall, 10 (48%) out of 21 small mammals were infested by ticks, as follows: three *Didelphis marsupialis* (30 *Amblyomma* sp. larvae, and 2 *Ixodes* sp. nymphs); three *Monodelphis domestica* (three *Ixodes* sp. nymphs) and four *Dasyprocta* sp. rodents (three *Amblyomma* sp. larvae, three *Ixodes* sp. nymphs and 16 *Ixodes* sp. larvae), making a total of 57 tick specimens. Two of the collected nymphs were molecularly identified as *Ixodes bocatorensis,* based on mitochondrial 16S rRNA gene partial sequences. Since all eight *Ixodes* sp. nymphs collected in this study presented the same morphotype, the molecular identification of two specimens as *I. bocatorensis* supports the identification of all collected nymphs as belonging to this same tick species. This study provides host records for immature stages of *I*. *bocatorensis* for the first time. Additionally, a brief morphological description of the *I. bocatorensis* nymph is provided.

## Resumo

Pesquisa sobre carrapatos que se alimentam de pequenos mamíferos é importante, porque eles podem ser vetores de bioagentes patogênicos que infectam animais e seres humanos. Neste estudo, pequenos mamíferos foram capturados na Área de Proteção Ambiental do Maracanã, no bioma Amazônia Oriental (São Luís, Maranhão), e foram inspecionados visualmente para detectar a presença de ectoparasitas. No total, 10 (48%) dos 21 pequenos mamíferos foram infestados por carrapatos, como segue: três *Didelphis marsupialis* (30 larvas de *Amblyomma* sp. e duas ninfas de *Ixodes* sp.); três *Monodelphis domestica* (três ninfas de *Ixodes* sp.) e quatro roedores *Dasyprocta* sp. (três larvas de *Amblyomma* sp., três ninfas de *Ixodes* sp. e 16 larvas de *Ixodes* sp.), totalizando 57 espécimes de carrapatos. Duas das ninfas coletadas foram identificadas molecularmente como *Ixodes bocatorensis*, com base em sequências parciais do gene mitocondrial 16S rRNA. Como todas as oito ninfas de *Ixodes* sp. coletadas neste estudo apresentavam o mesmo morfotipo, a identificação molecular de dois espécimes como *I*. *bocatorensis* suporta a identificação de todas as ninfas de *Ixodes* coletadas como pertencentes a essa mesma espécie de carrapato. Este estudo fornece registros de hospedeiros para estágios imaturos de *I*. *bocatorensis* pela primeira vez. Além disso, é fornecida uma breve descrição morfológica da ninfa de *I*. *bocatorensis*.

The genus *Ixodes* Latreille, 1795, represented by nearly 280 species, is the only tick genus that has been reported in all continents of the world, including Antarctica (Guglielmone et al., 2023; Apanaskevich et al., 2022). In Brazil, the genus *Ixodes* is currently represented by 12 species, listed in the following chronological order of description of the taxon: *Ixodes fuscipes* Koch, 1844; *Ixodes loricatus* Neumann, 1899; *Ixodes spinosus* Neumann, 1899; *Ixodes amarali* Fonseca, 1935; *Ixodes luciae* Sénevet, 1940; *Ixodes longiscutatus* Boero, 1944; *Ixodes schulzei* Aragão & Fonseca, 1951; *Ixodes lasallei* Méndez and Ortiz, 1958; *Ixodes paranaensis* Barros-Battesti, Arzua, Pichorim & Keirans, 2003; *Ixodes bocatorensis* Apanaskevich & Bermúdez, 2017; *Ixodes catarinensis* Onofrio & Labruna, 2020; and *Ixodes rio* Apanaskevich & Labruna, 2022 (the later species was previously reported in Brazil as *Ixodes auritulus* Neumann, 1904) (Onofrio et al., 2020; Apanaskevich et al., 2022; Labruna et al., 2024).

*Ixodes bocatorensis* was recently described in Bocas del Toro Province in Panama, and reported in South American countries, especially in the northern region of the continent, i.e., Brazil, Colombia and Venezuela (Apanaskevich & Bermudez, 2017; Onofrio et al., 2020). The adult stages (female and male) of *I*. *bocatorensis* have been collected from wild mammals of the orders Rodentia (Cuniculidae, Dasyproctidae), Perissodactyla (Tapiridae) and Xenarthra (Myrmecophagidae). These wild mammals are widely distributed on the continent; hence, it is likely that the distribution of *I. bocatorensis* is wider in the Amazon region (Michalski et al., 2015; Fernandes et al., 2023). However, the immature stages (larvae and nymphs) of this tick species have not been described, and their hosts are unknown (Apanaskevich & Bermudez, 2017; Martins et al., 2024). In addition to this paucity of information in the scientific literature, there is a lack of knowledge about the ecology and medical and veterinary importance of this tick species.

Between January 2018 and October 2020, ticks were collected from small mammals captured using live traps (Sherman and Tomahawk-like models) baited with peanut candy, sardines, and oats, as previously described (Szabó et al., 2013; Serpa et al., 2021), in the Maracanã Environmental Protection Area (EPA) (02° 24’09”S and 44° 29’47”W), located in São Luís municipality, state of Maranhão, eastern Brazilian Amazon (IMESC, 2020). The captured animals were anesthetized via intramuscular with injectable ketamine (50 mg/kg) and xylazine (20 mg/kg) according to Serpa et al. (2021), and were identified to species following Bonvicino et al. (2008) and Rossi et al. (2012).

Tick specimens were manually collected from small mammals using tweezers, and stored in individual microtubes containing 70% alcohol for later screening, processing and taxonomic identification based on specific literature for the genera *Ixodes* and *Amblyomma* (Barros-Battesti et al., 2024; Nava et al., 2017). To confirm the morphological identification of the genus *Ixodes*, two nymphal ticks were subjected to molecular testing based on PCR amplification of a 460 bp fragment of the tick mitochondrial 16S rRNA gene, following the protocol described by Mangold et al. (1998). The PCR products were purified and sequenced using the Sanger method.

A total of 21 small mammals were captured, being 17 marsupials (six *Didelphis marsupialis*, and eleven *Monodelphis domestica*) and four rodents of the genus *Dasyprocta.* Overall, 10 (48%) small mammals were infested by ticks, as follows: three *D. marsupialis* (30 *Amblyomma* sp. larvae, and 2 *Ixodes* sp. nymphs); three *M. domestica* (three *Ixodes* sp. nymphs) and four *Dasyprocta* sp. rodents (three *Amblyomma* sp. larvae, three *Ixodes* sp. nymphs and 16 *Ixodes* sp. larvae), making a total of 57 tick specimens. Partial sequences (411 bp) of the mitochondrial 16S rRNA gene were generated from two *Ixodes* nymphs (one from *M. domestica,* one from *D. marsupialis*), which consisted of a single haplotype that by BLAST analysis showed 99% identity (100% query cover) with *I. bocatorensis* sequences (accession numbers MN727316, MN727315) from Manaus, state of Amazonas, western Brazilian Amazon. Since all eight *Ixodes* sp. nymphs collected in this study presented the same morphotype, the molecular identification of two specimens as *I. bocatorensis* supports the identification of all collected nymphs as belonging to this same tick species.

The 16S rRNA gene haplotype of *I. bocatorensis* generated in this study has been deposited in GenBank under the accession number PV223868. In addition, two *I. bocatorensis* nymphs removed from *M. domestica* in Maracanã EPA were deposited in the tick collection “Coleção Nacional de Carrapatos Danilo Gonçalves Saraiva” (CNC) under the accession number CNC-4284.

The present data expand the distribution range of *I. bocatorensis* in the easternmost part of Brazil’s Legal Amazon region, revealing new associations between marsupials (*D. marsupialis* and *M. domestica*) and *I. bocatorensis* nymphs. To the best of our knowledge, this is the first report of any immature stage of this tick species, for which previous records relied exclusively on adult stages. Previous studies have reported collecting both sexes of *I. bocatorensis* on *Bradypus*, *Cuniculus*, *Dasyprocta* and *Tapirus* in Panama, Colombia and Venezuela (Apanaskevich & Bermúdez, 2017). *Ixodes bocatorensis* has been reported parasitizing rodents (*Dasyprocta* sp. and *Dasyprocta leporina*) and Xenarthra (*Bradypus tridactylus*, *Cyclopes didactylus* and *Tamandua tetradactyla*) in Manaus, state of Amazonas, Brazil (Onofrio et al., 2020).

The small mammals (*D. marsupialis*, *M. domestica* and *Dasyprocta* sp.) reported here are part of the most diverse groups of animals recorded in the Cerrado and Amazon biomes in the state of Maranhão (Ávila-Pires, 1989, 1992; Oliveira et al., 2007, 2011; Vieira & Oliveira, 2020). The presence of *I. bocatorensis* expands its distribution to the eastern Amazon, since the only previous reports in Brazil were from the western Brazilian Amazon (Onofrio et al., 2020). Studies on parasite-host interactions in wild animals are essential for conservation programs, contributing to shed light on the negative impacts of these parasites on biodiversity, and hence, on public health, and acting as indicators of ecosystem quality.

**Figure 1:**
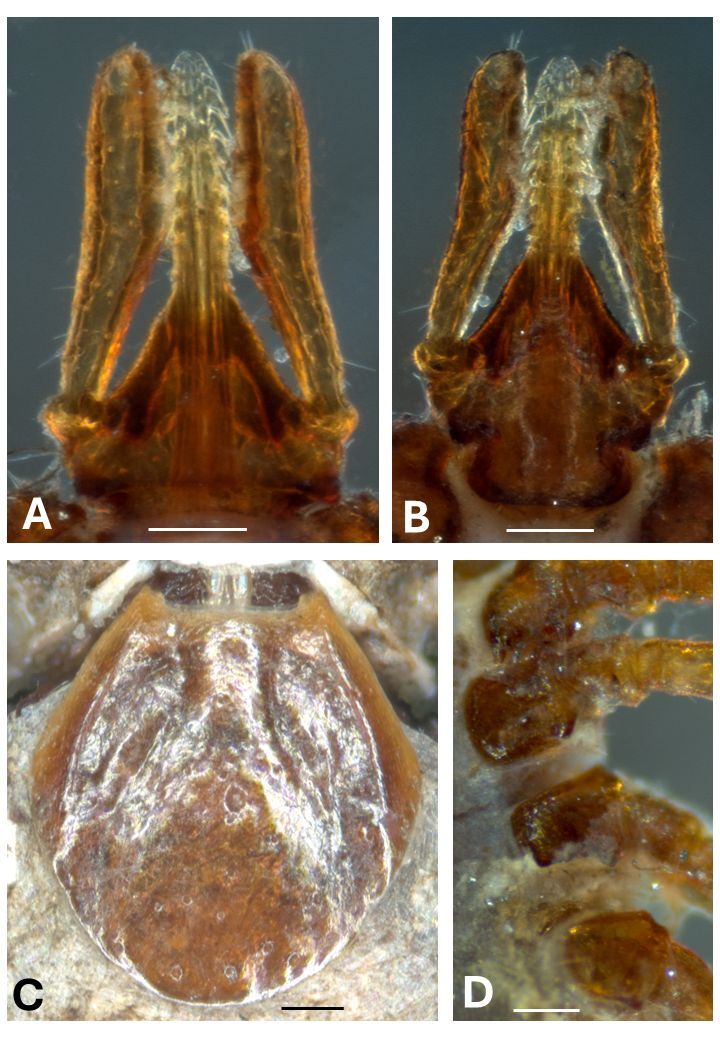
Light microphotographs of the nymph of *Ixodes bocatorensis* collected on *Monodelphis domestica* in São Luis, state of Maranhão, Brazil. (A) Dorsal gnathosoma. (B) Ventral gnathosoma. (C) Scutum. (D). Coxae I-IV. Scale bars: 100 µm.

**Description of the Nymph of *Ixodes bocatorensis* (Figure 1)**

The following description is based on two partially engorged nymphs, deposited under CNC-4284. The present two nymphs were observed under a stereomicroscope (Zeiss Stemi SV 11, Zeiss, Munich, Germany), where they were measured, and light photographs were taken using the software ZEN 2 core. All measurements are given in micrometers (the remaining nymphs of *I. bocatorensis* collected in this study were not available for description because they have been destroyed for DNA extraction for other purposes).

Gnathosoma length from palpal apices to cornua apices: 467–512; width of basis capituli: 275–287. Dorsal basis capituli subtriangular (Figure 1A), posterior margin nearly straight; cornua moderate, broadly triangular with blunt apices. Ventral basis capituli (Figure 1B) narrowed posterior to auriculae, with straight posterior margin and lateral angles rounded; short triangular auriculae. Palpi elongate and slender, article I strong. Hypostome moderately long, narrowly rounded apically, arising from a prominent anterior extension of the basis; dental formula 3/3 for anterior third, then 2/2 to base, the external row with larger teeth. Scutum (Figure 1C) length 655–695 (measured from scapular angle to posterior margin), width 586–624; few large punctations scattered distributed; cervical grooves distinct, shallow and reaching posterior third; lateral carinae as moderately sharp ridges; scapulae prominent. Legs (Figure 1D) moderately long, slender; coxae I with two spurs, the internal longer and pointed; a small triangular external spur on coxae I-IV; internal spur on coxae II-III indicated by a ridge like angle.
